# Perinatal outcomes of pregnancy in the fifth decade and beyond– a comparison of very advanced maternal age groups

**DOI:** 10.1038/s41598-020-58583-6

**Published:** 2020-02-04

**Authors:** Anat Schwartz, Ariel Many, Udi Shapira, Michal Rosenberg Friedman, Yariv Yogev, Tomer Avnon, Swati Agrawal, Shiri Shinar

**Affiliations:** 1 Department of Obstetrics and Gynecology, Lis Maternity Hospital, Sourasky Medical Center, Tel Aviv, Israel; 2 Sackler School of Medicine, Tel Aviv University, Tel Aviv, Israel; 3 Department of Obstetrics and Gynecology, Mount Sinai Hospital, Toronto, ON Canada

**Keywords:** Health care, Pregnancy outcome

## Abstract

To study the effect of very advanced maternal age on perinatal outcomes. A retrospective cohort study of women aged 45 years and above, who delivered ≥22 weeks of gestation in a single tertiary center between 1/ 2011 and 12/ 2018. Maternal and neonatal outcomes were compared between women ≥50 years and women of 45–49 years at delivery. Of 83,661 parturients, 593 (0.7%) were 45–49 years old and 64 (0.07%) were ≥50 years old. Obstetrical characteristics were comparable, though the rate of chronic hypertension and preeclampsia with severe features were greater in women ≥50 years (6.2% vs 1.4%, p = 0.04, 15.6% vs 7.0%, p = 0.01, 95% CI 0.19–0.86, respectively). Elective cesarean deliveries were independently associated with advanced maternal age ≥50 (OR 2.63 95% CI 1.21–5.69). Neonatal outcomes were comparable for singletons, but rates of ventilatory support and composite severe neonatal outcomes were higher in twin pregnancies of women ≥50 years (42.8% vs 13.5%, p = 0.01, and 21.4% vs 4.0%, p = 0.03, respectively). Healthy women ≥50 have higher elective cesarean rates, despite similar maternal and neonatal characteristics.

## Introduction

Tremendous advances in artificial reproductive technologies (ART) and egg donation in the last 2–3 decades enable perimenopausal, and even postmenopausal women aged 50 years and above to conceive and deliver. Subsequently, despite the decreasing overall birth rates in the general population from 2006 to 2017, there has been a persistent rise in birth rates in women older than 30 years of age, with the greatest rise of 19% seen in women in their early forties^1^. For women of 45 years and above an all-time new birth rate peak of 0.9 per 1,000 births was recorded in 2016^2,3^. This shift in childbearing years is expected to continue to grow with increasing ART availability.

Historically, advanced maternal age was defined as women ≥35 at the time of delivery^4^ and studies on pregnancy outcome compared women in this age group to women younger than 35^5,6^. With the shift in childbearing years from the third and fourth decades to the fifth decade and beyond, two new terminologies arose - “very advanced maternal age” for women ≥40 years and “extremely advanced maternal age” for women ≥45 years^7–9^.

While it has been established that pregnancy outcomes in women above 45 are generally poorer than those of women in their third or fourth decade of life^10–12^, there is scarce data regarding the difference between pregnancy outcomes in the sixth and seventh decades of life and those in the fifth decade of life^1,7,9,10,13^. Studies investigating this difference have generally regarded women above 45 as one group^1,6^. Additionally, it was suggested that poorer pregnancy outcomes are the result of pre-existing medical disorders and are not independently associated with maternal age^14^. Current data on pregnancies in women above 50 is mainly restricted to relatively small cohorts and study designs that lack comparison groups^14,15^. Moreover, outcomes previously studied were primarily maternal^1,9,13^, with paucity of data on neonatal outcomes. Thus, we aimed to determine whether women of 50 years and beyond are at higher risk for adverse maternal and neonatal outcomes and to assess how plurality affects adverse outcomes in this age group in comparison to women of 45–49 years of age.

## Methods

This was a retrospective cohort study at a single tertiary university affiliated medical center of women above the age of 45 at delivery and their neonates, who delivered at or beyond 22^0/6^ weeks of gestation from Jan 1, 2011 to Dec 31, 2018. For the purpose of comparison, the cohort was divided into 2 groups: women aged 50 and above (study group) and women at 45–49 years of age (control group) at the time of delivery. Maternal pregnancy and delivery outcomes were assessed. Neonatal outcomes were analyzed separately for singleton and for twin pregnancies in these two maternal age groups. The study was approved by our institutional review board (The Tel-Aviv Sourasky medical center IRB 0284-08-TLV) and was conducted in accordance with the STROBE guidelines and for observational cohort studies. Informed consent waived by our IRB due to the retrospective nature of the study and the fact that all data was deidentified and anonymous.

Our center has one of the largest obstetrical units in Israel and is situated in a central metropolitan, serving a very heterogenous population of varied ethnic backgrounds and socioeconomic status. Data were extracted from computerized delivery room logbooks and neonatal intensive care unit databases that are updated in real time in our institution. Parameters of interest included demographics and medical history (particularly preexisting cardiovascular diseases, diabetes mellitus and chronic hypertension), obstetrical history and labor and delivery characteristics of the index gestation. Maternal and neonatal outcomes were assessed until the time of discharge from hospital. A subgroup analysis of neonatal outcomes during the hospitalization period was performed for singleton and twin pregnancies in the two maternal age groups. For these, ventilatory support was defined as continuous positive airway pressure (CPAP) or mechanical ventilation. Composite severe neonatal morbidity was defined as one or more of the following: intraventricular hemorrhage, necrotizing enterocolitis, asphyxia (defined by Sarnat staging for hypoxic-ischemic encephalopathy) or perinatal death (defined as stillbirth or neonatal death from 22 weeks of gestation to 7 days of life^16–18^.

Pre-gestational diabetes and gestational diabetes mellitus, as well as hypertensive complications of pregnancy, were defined according to criteria of the American College of Obstetricians and Gynecologists (ACOG)^19,20^. Preeclampsia with severe features was defined according to the latest ACOG Task Force guidelines on Hypertension in Pregnancy from 2013^20^. Small and large for gestational age were defined as birthweight below 10th percentile and above 90th percentile, respectively, according to national liveborn infant birthweight curves^21^.

All statistical analyses were performed using SPSS software (SPSS version 25, IBM, Chicago). Comparison between the two groups was performed with student *t* test for continuous variables that are normally distributed and with Mann-Whitney rank sum test for continuous variables that are not normally distributed. Chi-square and Fisher’s exact test were used for categorical variables where appropriate. Binary logistic regression was performed for all possible known confounders. A p-value < 0.05 was considered statistically significant.

## Results

During the study period, 83,661 women delivered in our hospital. Of these, 593 (0.7%) women were 45–49 years old and 64 (0.07%) were above 50 years of age (range 50–58) at the time of delivery. Pre-gestational BMI, weight gain during pregnancy, pre-gestational diabetes and aspirin use in pregnancy were comparable in both groups (Table 1). Though the frequency of IVF pregnancies was high in the entire cohort, it was significantly higher among women >50 years (p < 0.01), all of whom required IVF to conceive. The prevalence of chronic hypertension was significantly higher in women above 50 compared to women of 45–49 years of age (6.2% vs 1.4%, respectively, p = 0.04).

**Table 1:** Maternal demographic and obstetric characteristics in women ≥50 compared to women of 45–49 years of age.

	≥50 yrs (n = 64)	45–49 yrs (n = 593)	P-value
Age at delivery (yrs)	52.4 (2.2)	46.5 (1.2)	<0.01
Primiparity	36 (56.2)	296 (49.9)	0.33
Previous CS^a^	13 (46.4)	111 (37.4)	0.34
IVF pregnancy	64 (100)	384^b^ (77.7)	<0.01
Multiple gestation	7 (10.9)	74 (12.5)	0.72
Pre-gestational BMI (kg/m^2^)	22.7 (6.6)	24.5 (6.7)	0.04
Weight gain in pregnancy (kg)	11.4 (5.7)	11.4 (6.0)	1.00
Pre-gestational diabetes	2 (3.1)	11 (1.8)	0.62
Chronic hypertension	4 (6.2)	11 (1.8)	0.04
Aspirin use in pregnancy	8 (16)	38 (6.4)	0.07

yrs years; CS cesarean section; BMI body mass index.

^a^Calculated from total number of multiparous women.

^b^Reported for 494 women in the control group.

Data are presented as n (%) or mean (SD) where appropriate. Categorical data were compared using Fisher’s exact test. Continuous variables were compared using student’s t-test.

Significant differences were found in maternal outcomes of women above 50 compared to women of 45–49 years of age (Table 2). While there were no differences in rates of gestational hypertension or preeclampsia without severe features, preeclampsia with severe features was more prevalent in the study group than in the control group (15.6% vs 7.0%, respectively, p = 0.01). Additionally, the rate of elective cesarean delivery was higher in the former (53.1% vs. 35.6%, respectively, p < 0.01) as was the overall cesarean delivery rate (87.5% vs. 71.1%, respectively, p < 0.01). In multivariate logistic regression analysis adjusting for multiple gestation, previous cesarean, pregestational BMI and preeclampsia, cesarean deliveries were independently associated with advanced maternal age above 50 years of age (OR 3.00 95% CI 1.29–6.98, Table 3). No other maternal outcome was independently associated with advanced maternal age above 50 when compared to women of 45–49 years of age. Specifically, after adjusting for chronic hypertension, aspirin use in pregnancy, pregestational and gestational diabetes and multiple pregnancy, women above 50 years of age were not at a greater risk for preeclampsia (OR 1.57, 95% CI 0.76–3.24).

**Table 2:** Maternal outcomes in women ≥50 compared to women of 45–49 years of age.

	≥50 yrs (n = 64)	45–49 yrs (n = 593)	P-value
Hypertensive disease	12 (18.7)	72 (12.1)	0.13
Preeclampsia with severe features	10 (15.6)	42 (7)	0.01
Diabetes in pregnancy	11 (17.2)	118 (19.8)	0.6
Cesarean Delivery	57 (87.5)	422 (71.1)	<0.01
Elective CS	34 (53.1)	211 (35.6)	<0.01
Urgent CS	22 (34.3)	211 (35.6)	0.84
Length of hospitalization (days)	6.1 (6.6)	4.7 (6.2)	0.09
Postpartum blood transfusion	2 (3.1)	27 (4.5)	0.76
ICU admission	2 (3.1)	6 (1)	0.17

CS cesarean section; ICU intensive care unit.

Hypertensive disease was defined as gestational hypertension, superimposed preeclampsia or preeclampsia without severe features; Diabetes in pregnancy was defined as preexisting diabetes or gestational diabetes.

Data are expressed as mean ( ± SD), n(%) or median (interquartile ranges) where appropriate. Categorical data were compared using Fisher’s exact test.

**Table 3:** Maternal outcomes in women ≥50 compared to women of 45–49 years of age - multivariate analysis.

Outcome	Odds ratio (95% CI)
Cesarean delivery	3.00 (1.29–6.98)
Preeclampsia	1.57 (0.76–3.24)

Cesarean delivery was adjusted for previous cesarean delivery, preeclampsia, pre-gestational BMI and multiple gestation.

Preeclampsia was adjusted for use of aspirin in pregnancy, chronic hypertension, pre-gestational diabetes, gestational diabetes, and multiple gestation.

For singleton pregnancies, neonatal outcomes were comparable in the study and control groups, but differed for multiples (Table 4). For multiples, rates of ventilatory support and composite severe neonatal outcomes were significantly higher in the study group (42.8% vs 13.5%, p = 0.01, and 21.4% vs 4.0%, p = 0.03, respectively), as was length of NICU stay (58.1 (31.3) days vs 28 (18.7) days, p < 0.01). These findings may be explained by an earlier gestational age at delivery in women above 50 years of age, a difference that approached statistical significance (33.7 vs 35.3 weeks, p = 0.05). In multivariate logistic regression analysis of the entire neonatal cohort no neonatal outcome was independently associated with advanced maternal age above 50 when compared to women of 45–49 years of age (Table 5).

**Table 4:** Neonatal outcome in women ≥50 compared to women of 45–49 years of age.

Singleton gestation			
	≥50 yrs (n = 57)	45–49 yrs (n = 519)	P-value
Gestational age at delivery	37.7 (1.8)	38.1 (2.1)	0.17
<32 week	1 (1.7)	8 (1.5)	0.61
<34 week	2 (3.5)	17 (3.3)	0.57
<37 week	10 (17.5)	59 (11.4)	0.17
Neonatal birthweight <10%	5 (8.7)	34 (6.5)	0.57
Neonatal birthweight <5%	1 (1.7)	19 (3.7)	0.71
NICU admission	7 (12.2)	48 (9.2)	0.45
Length of hospitalization in NICU (days)	16.4 (4.5)	21 (20.9)	0.10
Neonatal hypoglycemia	1 (1.7)	17 (3.3)	0.71
Ventilatory support	4 (7.0)	16 (3.0)	0.12
Cord pH <7.1 at delivery	0	11 (4.4)	0.38
Apgar <7 at 5 min	0	7 (1.3)	0.48
Perinatal mortality	0	5 (0.9)	0.62
Composite severe neonatal outcome	1 (1.7)	7 (1.3)	0.56
**Multiple gestation**			
	**≥50 yrs (n = 14)**	**45–49 yrs (n = 148)**	**P-value (95% CI)**
Gestational age at delivery	33.7 (4.2)	35.3 (2.7)	0.05
<32 week	2 (14.2)	16 (10.8)	0.48
<34 week	6 (42.8)	32 (21.6)	0.09
<37 week	8 (57.1)	80 (54)	0.82
Neonatal birthweight <10%	1 (7.1)	18 (12.1)	0.70
Neonatal birthweight <5%	0	10 (6.7)	0.60
NICU admission	6 (42.8)	48 (32.8)	0.55
Length of hospitalization in NICU (days)	58.1 (31.3)	28.0 (18.7)	<0.01
Neonatal hypoglycemia	0	14 (9.4)	0.37
Ventilatory support	6 (42.8)	20 (13.5)	0.01
Perinatal mortality	0	3 (2)	0.30
Cord pH <7.1 at delivery	0	1 (2.3)	0.80
Apgar score <7 at 5 min	1 (7.1)	5 (3.4)	0.42
Composite severe neonatal morbidity	3 (21.4)	6 (4)	0.03

Data are presented as n(%) or mean (±SD) where appropriate. Categorical data were compared using Fisher’s exact test.

NICU Neonatal intensive care unit.

Ventilatory support was defined as continuous positive airway pressure (CPAP) or invasive ventilation.

Perinatal mortality was defined as stillbirth or neonatal death from 22 weeks’ gestation to 7 days of life.

Asphyxia was defined by Sarnat staging for hypoxic-ischemic encephalopathy^18^.

Neonatal birthweight centile was defined according to national neonatal growth curves^21^.

Composite severe neonatal morbidity was defined as one or more of the following: perinatal mortality, intraventricular hemorrhage, necrotizing enterocolitis or asphyxia.

**Table 5:** Neonatal outcomes in women ≥50 compared to women of 45–49 years of age - multivariate analysis.

Outcome	Odds ratio (95% CI)
NICU admission	1.00 (0.38–2.64)
Ventilatory Support	2.61 (0.86–7.92)
Composite severe neonatal morbidity	1.10 (0.34–3.51)
SGA <10%	1.00 (0.41–2.45)

NICU neonatal intensive care unit.

Ventilatory support was defined as continuous positive airway pressure (CPAP) or invasive ventilation.

Composite severe neonatal morbidity was defined as one or more of the following: perinatal mortality, intraventricular hemorrhage grades 1–4, necrotizing enterocolitis or asphyxia.

All the above neonatal outcomes were adjusted for gestational age at delivery, mode of delivery and SGA.

## Discussion

In the present study we found that women of 50 years of age and above were at a significantly higher risk for elective cesarean delivery, compared to women of 45–49, regardless of their co-morbidities and obstetrical history. They were also at an increased risk for preeclampsia with severe features, a risk that was likely attributed to higher rates of chronic hypertension compared to their younger counterparts. Lastly, while neonatal outcomes for singletons were comparable in both age groups, they were poorer for twins of older women.

Our comparison groups were alike in their demographic, obstetrical characteristics and medical history, except for higher rates of chronic hypertension in women above 50 years of age. These results differ from previously reported morbidity rates in women of advanced age, which showed higher rates of chronic hypertension, but also higher rates of pre-gestational diabetes mellitus^10^ and gestational diabetes^9^. We found that maternal outcomes were mostly favorable, but rates of preeclampsia with severe features were significantly higher among women above 50 years of age. Though this risk grew substantially with increasing age, it was not independently associated with age, but more likely driven by underlying medical conditions, such as chronic hypertension. This finding highlights the fact that women of 50 years of age and above are not at greater risk by virtue of their age, if they are healthy and do not have pre-existing medical conditions, that are associated with poorer maternal outcome. This important finding is supported Carolen *et al*., who demonstrated favorable outcomes in older women without pre-existing hypertension^7^, as well as by Osmundson *et al*., who found severe maternal morbidity in women with pre-existing hypertensive disorders^13^. Thus, preconception counseling with screening for hypertensive diseases, should be considered, and women with chronic hypertension should be counseled as to their greater risk of adverse pregnancy outcomes.

Multiple studies have shown that rates of cesarean delivery increase with advancing maternal age^9,10,13,22^ above 40. When assessing this risk in parturients above the age of 50 with a singleton pregnancy, the risk was found to range between 68%^12^ to 100%^10^, with more recent literature, quoting a rate of 85%^9^, similar to that of women between 45–49 years. Many of these studies did not distinguish between pre-labor cesarean delivery and intrapartum cesareans. Our study shows that while emergency cesareans, the majority of which were intrapartum, were no more frequent in women above 50 than at 45–49 years, elective cesarean delivery rates were significantly higher with advancing maternal age, accounting for 53% of all cesareans. This finding supports the independent association between advancing maternal age and the higher incidence of cesarean delivery. Though we did not stratify for cesarean section indication, we believe that this high rate may be driven by the provider’s and patient’s preference not to labor, assuming that this would be the patient’s last delivery^9,23^. It may also highlight an obstetric culture of not laboring women of advanced maternal age^22^. This is despite the findings that a successful trial of labor among women ≥50 years of age is estimated at 74%^13^.

Multiple gestations were common in our cohort, accounting for 12.5% of mothers ≥45 and 11% of mothers ≥50 years of age. This finding, likely resulting from the high incidence of IVF treatment in this age group, is supported by Osmundson *et al*., who also showed high rates of multiples in women ≥50^13^. Like previous studies that documented an increased risk for preterm deliveries among women of advanced and extremely advanced maternal age^7,9,10,13,24^, rates of prematurity were comparably high among women of 50 and beyond and those of 45–49 years of age. Higher rates of ventilatory support and severe composite neonatal morbidity among twins of women aged 50 and above may be explained by the earlier gestational age at delivery. These complications, along with prematurity are reflected in the longer NICU stays in the neonates of these women. Additionally, higher rates of ventilatory support may be related to higher rates of cesarean delivery in women above 50, that may have resulted in transient tachypnea of the newborn (TTN)^23,25^. This difference was not maintained in multivariate analysis, emphasizing that maternal age does not independently affect neonatal morbidity.

The main strengths of this study rely on the fact that only women who delivered at 22 weeks of gestation or greater were included, enabling us to focus on maternal complications associated with peri-viable and viable pregnancies and to assess delivery outcomes. Additionally, the unique comparison of our cohort to a large control group of women of 45 to 49 years of age, is of clinical relevance. Caregivers may often regard the risks and complications of women ≥50 years as similar to those of women in their fifth decade of life, a group close in age but with much greater representation in the literature. This study is of the few existing studies that specifically compares women >50 years of age, showing that while they do not differ substantially in their risk profile and pregnancy outcome, their pregnancies are still perceived as riskier, and therefore, are at a significantly higher risk for elective, possibly not obstetrically indicated, cesarean sections. Lastly, as nearly all pregnancies beyond the age of fifty are a result of IVF treatments, as is evident from our cohort, multiple gestations are frequently overrepresented in this population. Our sub-analysis of outcomes in twins is thus valuable, despite its small numbers, since it provides support that the neonatal outcome is not independently affected by extreme maternal age, but by complications inherent to multiple gestations and maternal health.

A significant limitation of our study is that we could not study the effect of egg donation on pregnancy outcomes. While it is most plausible that nearly all pregnancies beyond the age of fifty were conceived with donor egg, this could not be fully ascertained by maternal questioning, as it is often underreported^24^. Secondly, we were unable to perform multivariate analysis to study the effect of maternal age on neonatal outcomes in twins, due to a small sample size. Thirdly, we did not differentiate between spontaneous pre-term births and iatrogenic preterm deliveries. Our extremely high rate of elective cesarean deliveries in women above 50 suggests that the latter was most likely more prevalent. Fourthly, we were unable to adjust for smoking and low socioeconomic status, factors that are known to adversely affect pregnancy outcome^15,26^. This is because the former was not available and the latter was most probably underreported in our cohort, with a much lower incidence than previously reported in the general pregnant population^27^. Lastly, our cohort consisted of mostly normal pre-gestational BMI women. As such, there is a theoretical concern regarding generalizability of our results in pregnancies complicated by obesity.

We conclude that pregnancy at 50 years and above is associated with a significantly increased risk of cesarean delivery, beyond that of women of 45–49 years of age. While favorable maternal and neonatal outcome can be expected in healthy women >50, those with pre-existing medical conditions are at significantly higher risk for preeclampsia with severe features. The main predictor of outcome is, therefore, maternal health and not maternal age. As such, pre-pregnancy counseling should be offered to these women with screening for hypertensive diseases. Further studies are needed to assess vaginal delivery outcomes in healthy women of extremely advanced maternal age.
